# Pu-erh Tea Extract Treatment Could Be an Efficient Way to Enhance the Yield and Nutritional Value of Soybean Sprout

**DOI:** 10.3390/molecules25173869

**Published:** 2020-08-25

**Authors:** Jeong-Ho Kim, Yong-Han Yoon, Il-Doo Kim, Sanjeev Kumar Dhungana, Dong-Hyun Shin

**Affiliations:** 1 Department of Green Technology Convergence, Konkuk University, Chungju 27478, Korea; hoya1209@kku.ac.kr (J.-H.K.); yonghan7204@kku.ac.kr (Y.-H.Y.); 2 International Institute of Research & Development, Kyungpook National University, Daegu 41566, Korea; ildookim@hanmail.net; 3 Department of Southern Area Crop Science, National Institute of Crop Science, Rural Development Administration, Miryang 50424, Korea; sanjeevdhungana@yahoo.com; 4 School of Applied Biosciences, Kyungpook National University, Daegu 41566, Korea

**Keywords:** antioxidant potential, nutrient, Pu-erh tea, soybean sprout, yield

## Abstract

Soybean sprouts are one of the most inexpensive and nutritious food items that can be easily grown year-round. Several studies have been conducted to increase their yield and nutritional values. This study was carried out to examine the effects of Pu-erh tea extracts on the production and nutrients content of soybean sprouts. Soybean seeds were soaked in 1%, 2%, or 3% (*w*/*v*) tea extracts, or tap water, before keeping for sprout cultivation; the sprout samples were named PE-1, PE-2, PE-3, and the control, respectively. The sprout yields were increased by up to 17% in PE-2 and PE-3 than in the control. The vitamin C, total free amino acid, total mineral, total isoflavone, total polyphenol, and flavonoid contents as well as the antioxidant potentials of the tea extract-treated sprouts were higher than those of the control. The results indicated that pre-soaking soybean seeds in 2% Pu-erh tea extracts could offer an easy, inexpensive, and efficient way to improve the yield and nutritional value of soybean sprouts.

## 1. Introduction

Soybeans are good sources of protein, fat, carbohydrates, and other phytochemicals that are essential for good health. However, soybean seeds also contain several anti-nutritional factors (ANFs) that may induce malnutrition situations and affect the human population, particularly in the developing world [1]. Germination can lessen the negative effects of ANFs and improve the nutritional values of non-germinated seeds [2,3,4,5]. Not only are the contents of existing nutrients modified, but new components are also released due to germination [6]. Soybean sprouts are generally prepared in a week and can be grown using simple and inexpensive technologies, which are additional benefits in their production. In addition, soybean sprouts can be produced for year-round use. The nutrients such as γ-aminobutyric acid (GABA), lysine, vitamin E, dietary fiber, niacin, magnesium, vitamin B1, and vitamin B6 are significantly increased after germination [6]. Hence, the production of high-quality soybean sprouts with an increased yield is very important.

Several efforts have been made to improve the nutritional and functional properties of grains in the form of sprouts, including soybean, since germination is one of the efficient methods to enhance their food values [4]. An application of calcium chloride to seeds and sprouts increases the production and bioavailability of Zn, Fe, and Ca as well as GABA and the isoflavone content of soybean sprouts [7]. Similarly, the exposure of soybean seeds to ultrasound enhances the germination rate, sprout length, and GABA content of soybean sprouts [8]. Amino acids GABA and glycine are reported to be associated with learning and memory enhancement; stroke and neurodegenerative disease control; anxiety, sedation, and anticonvulsant relief; and muscle relaxation functions [9,10]. The high GABA-containing foods are found to have various bioactive functions, such as regulating blood cholesterol and pressure, decreasing insomnia and depression, and relieving pain [11]. Intake of GABA regulated blood pressure and inhibited sleeplessness and autonomic disorder observed during the menopausal or presenile period [12]. GABA also has a role in controlling diabetes [13]. The Pu-erh tea extract treatment significantly increased the amounts of many amino acids in soybean sprouts. Exposure to light increases the total phenolic and saponin content of soybean sprouts [14]. Gamma irradiation increases the microbial safety of soybean seeds and sprouts by reducing the risk of foodborne pathogens [15]; the spraying of zinc sulfate solution enhances the zinc content of soybean sprouts [16]; different light treatments manipulate the isoflavone contents of soybean sprouts [17]; the inoculation of bacterial strains affects the bioactive compounds and antioxidant potentials of soybean sprouts [18]; spraying grapefruit seed extract, chitosan, and phosphate buffer improves soybean sprout yield and inhibits sprout rot [19]; treatment with the medicinal plant ginseng increases the growth as well as the amino acid and organic acid content of soybean sprouts [20]. Soaking soybean seeds in persimmon fruit powder enhances the yield and nutritional value (vitamin C, isoflavone, and total phenolic content) of soybean sprouts [21]. The germination of brown rice in red onion solution increases the antioxidant capacity and GABA content as well as makes the rice slightly softer and stickier than that germinated in water [22]. Although the use of a few synthetic plant growth regulators, such as Indolbi and 6-benzylaminopurine, to enhance the yield and nutritional values of soybean sprouts are in practice [23], due to potential health hazards, consumers are reluctant to accept such synthetic chemical-treated food products. Since a few studies show that the use of different plant products such as persimmon fruit powder [21], brown seaweed [24], lacquer stem [25], and ginseng [20] can significantly enhance the yield and/or nutritional value of soybean sprouts, the exploration of natural products to enrich the food quality is of great significance.

Pu-erh tea is receiving increased attention due to its health benefits for a variety of hypolipidemic, antiobesity, antimutagenic, antioxidative, antitumor, free radical scavenging, and toxicity-suppressing activities [26]. So far, no reports on the effect of phytochemical-rich Pu-erh tea on soybean sprouts have been published. Considering the health benefits of Pu-erh tea and the economical as well as nutritional values of soybean sprouts, this study was conducted to investigate the effect of Pu-erh tea on the yield and nutritional values of soybean sprouts.

## 2. Results

### 2.1. Yield and Moisture and Vitamin C Contents

Pu-erh tea soaking significantly affected the sprout yields and vitamin C content of soybean sprouts; however, the moisture contents of the sprouts were not influenced (Table 1). Sprout yields of PE-1, PE-2, and PE-3 were increased by 9.5%, 16.8%, and 16.9%, respectively, compared to that of the control. The vitamin C contents of PE-2 (18.07 mg/100 g fresh weight, FW) and PE-3 (18.09 mg/100 g FW) were highest followed by PE-1 (16.90 mg/100 g FW) and the control (16.00 mg/100 g FW).

### 2.2. Color Value of Soybean Sprouts

Hunter’s color values of soybean sprouts were significantly increased by Pu-erh tea treatment (Table 2). The lightness and yellowness values of PE-1 (75.39 and 22.32) and PE-2 (75.73 and 22.36) were significantly higher that of PE-3 (73.38 and 21.02) and the control (71.90 and 17.73). The significantly highest greenness value was found in PE-1 (−1.41), followed by PE-3 (−1.17) and PE-2 (−1.12), and the control (−0.81).

### 2.3. Free Amino Acid Composition

As other physicochemical characteristics, the treatment of Pu-erh tea extracts significantly influenced the free amino acid profile of soybean sprouts (Table 3). A total of 27 free amino acids were detected, whereas the amounts of 10 amino acids were non-detectable. The numbers of essential, non-essential, other free amino acids were 8, 8, and 11, respectively. The amounts of all the essential and non-essential amino acids were significantly higher in the Pu-erh tea-treated soybean sprouts than in the non-treated control. The amount of GABA, which is also known as a component of brain foods, was also higher in the tea-treated samples than in the control. The ratio of essential to non-essential amino acids was also increased by the treatment. PE-3 (50.10 mg/g dry weight, DW) contained the highest amount of total free amino acid followed by PE-2 (50.05 mg/g DW), PE-1 (49.93 mg/g DW), and the control (46.13 mg/g DW).

### 2.4. Mineral Content

Soaking of seeds in Pu-erh tea extracts also increased the minerals content of soybean sprouts (Table 4). K (16845.74–18180.18 mg/kg DW) and Mn (20.66–21.51 mg/kg DW) were the most and least abundant minerals in the sprout samples. Out of the eight minerals measured, six were higher in one of the tea extract-treated soybean sprouts than in the control. The Mn and Zn contents of the control were not significantly different from those of PE-3 and PE-2, respectively. The amount of total mineral content in the tea extract-treated soybean sprouts was higher than that in the control treatment.

### 2.5. Isoflavone Content

The amount of isoflavone components, except for genistein, was significantly increased in the sprouts with Pu-erh tea treatments (Table 5). Daidzin (321.41–401.21 mg/kg DW) and glycitein (10.11–15.71 mg/kg DW) were the most abundantly and scarcely contained isoflavones in the sprout samples. The amount of daidzin, genistin, glycitin, and glycitein was increased with the concentration of the tea extracts.

### 2.6. DPPH and SOD-Like Activities and Total Polyphenol and Flavonoid Contents

The free radical scavenging potentials of the soybean sprouts were determined through 1,1-diphenly-2-picrylhydrazyl (DPPH) and superoxide dismutase (SOD)-like activities, which were significantly increased with the tea extract treatments (Table 6). Similarly, the total polyphenol and flavonoid contents of the sprouts were also enhanced by the treatment (Table 6). Sprout samples PE-2 and PE-3 had the significantly highest DPPH free radical scavenging potential, whereas PE-1 had the highest SOD-like activity and total polyphenol content.

## 3. Discussion

Pu-erh tea extract treatment significantly influenced various physicochemical characteristics of soybean sprouts (Supplementary Table S1). Although the content of specific plant growth regulators in Pu-erh tea was not measured in the present study, it can be implied that some growth-promoting substances might have played a role in increasing the yield and nutrient content of the tea extract-treated soybean sprouts as in previous studies with the extracts of persimmon fruit powder [21], brown seaweed [24], lacquer stem [25], and ginseng [20]. This enhancement might be due to the absorption of various phytochemicals of Pu-erh tea [27,28] during seed-soaking and the subsequent physiological and biochemical mechanisms that took place during sprout growth [29].

The increment in the levels of hormones indoleacetic acid and gibberellin due to the presence of minerals such as calcium in the Pu-erh tea might have increased the yield and vitamin C content in the Pu-erh tea-treated soybean sprouts [7,30].

The color of a food product is a key factor in determining the willingness of consumers to pay for the product [31]. The tea extract treatment increased the yellowness and lightness of sprouts, which are some of the desirable features of soybean sprouts [32]. Although the reasons for variations in the color of sprouts were not well understood, Pu-erh tea treatment enhanced the color appearance of soybean sprouts.

The essential amino acids cannot be synthesized de novo by an organism at the required rate; thus, they must be supplied through diets. Both the content of essential amino acids and the ratio of essential to non-essential ones are important because the foods containing high ratios of essential to non-essential amino acids are considered ideal foods from the protein deposition point of view [13]. The amount of glutamic acid, a precursor for GABA synthesis [33], was increased in soybean sprouts after Pu-erh tea treatments. The high calcium content of Pu-erh tea might have played a role in the activation of diamine oxidase activity and thereby increased the GABA content in the tea-treated sprouts [7]. Similar results of increased total amino acid content, including GABA, were also found with lacquer-treated soybean sprouts [25].

The soaking of soybean seeds in the mineral-rich Pu-erh tea [30] extracts might have played a role in increasing the mineral contents of soybean sprouts as in the previous studies with zinc sulfate-applied soybean sprouts [16,34] and selenium-treated cereal sprouts [29]. Elements including, Fe, Zn, Cu, Ca, and Mg are most commonly lacking in human diets [35]. Minerals Mg, K, and Ca are beneficial against hypertension [36]; Fe contributes to oxygen transport, energy metabolism, mitochondrial respiration, DNA synthesis, and cellular growth and differentiation [37]; while Zn plays roles in growth, development, differentiation, DNA synthesis, RNA transcription, and cellular apoptosis [38]. The increments in the amount of various minerals with the tea extract treatment signify the usefulness of Pu-erh tea to enhance the mineral content of soybean sprouts.

The phytochemicals present in Pu-erh tea might have played roles to increase the isoflavone contents in the sprouts, although the exact mechanism was not clear. One of the reasons for elevated isoflavones might be owing to the increased phenylalanine content and thereby the isoflavone synthetase activities [7,39] due to the tea treatment. Soybean seed soaking with persimmon fruit powder extract also increased the isoflavone content of soybean sprouts [21]. Soy isoflavones are beneficial in preventing aging, cardiovascular diseases, and cancer because of their potential ability to scavenge the free radicals from the human body [40]. They have also shown both estrogenic and anti-estrogenic effects and are therefore beneficial against chronic diseases such as osteoporosis and hypercholesterolemia as well as for the alleviation of postmenopausal symptoms [41,42,43,44]. Results showed that Pu-erh tea can effectively enhance the isoflavones content in soybean sprouts.

Various kinds of reactive oxygen species (ROS), including hydrogen peroxide, hydroxyl radical, and singlet oxygen, cause oxidative damage in lipids, proteins, and DNA [45]. Higher levels of the ROS may propound risks to cells by lipids peroxidation, proteins oxidation, nucleic acids destruction, enzyme inhibition, the programmed cell death activation pathway, and eventually cell death [46,47]. The significantly elevated biosynthesis of antioxidants in the tea extract-treated samples might be owing to the high contents of elements such as calcium [30] and/or phenolic compounds [48,49] in Pu-erh tea. Similar results of high phenolic contents in the germinated brown rice were found when the rice was treated with high phenolic-containing onions [22,50,51] and soybean sprouts grown with lacquer treatment [25]. Phenolic compounds possess antioxidant potentials in vegetables and other foods [52,53]. Thus, the availability of total polyphenols and flavonoids might have contributed to the higher DPPH and SOD-like activities of the Pu-erh tea extract-treated soybean sprouts.

## 4. Materials and Methods

### 4.1. Chemicals and Experimental Materials

Folin–Ciocalteu phenol reagent, pyrogallol, and 1,1-diphenly-2-picrylhydrazyl (DPPH) were purchased from Sigma-Aldrich (Sigma-Aldrich Corp, St. Louis, MO, USA) and amino acid standards were obtained from Wako (Wako Pure Chemical Industries, Ltd., Osaka, Japan). All the chemicals used in this study were of analytical grade.

Soybean (*Glycine max* L.) cv. Sowonkong [54], obtained from the Agricultural Research and Extension Services, Gyeongsangbuk-do, Korea, was considered to produce sprouts. The mean 100-seed weight of the cultivar was 12 g. A typical wild Pu-erh tea produced in Yunnan province of China [55] was used in this study.

### 4.2. Cultivation of Soybean Sprouts

One kilogram of intact seeds, in three replicates, were washed with tap water and soaked in tap water or three different concentrations of Pu-erh tea extracts for 6 h. The three concentrations (1%, 2%, and 3%; *w*/*v*) of Pu-erh tea extracts were also prepared in tap water. The sprout samples were named control, PE-1, PE-2, and PE-3 for that soaked in tap water alone, 1%, 2%, and 3% Pu-erh tea extract, respectively. After soaking for 6 h, the seeds were thoroughly rinsed with tap water and put into 15-L plastic buckets and then covered with double-layered black landscape fabric to minimize light exposure during sprout growing [21]. The sprouts were sprinkled with tap water for 2 min every 3 h using two hoses of 1 cm diameter. Soybean sprouts were grown at 20 ± 1 °C for 6 days.

### 4.3. Measurement of Sprout Yield and Preparation of Sprout Powders

Sprout yields (fresh weight of soybean sprouts) in each batch were measured at 6 d. The yield of sprouts was calculated by deducting the weight of the empty bucket from the weight of each bucket containing sprouts. Sprout powders prepared from the sprouts with cotyledons, hypocotyl, and roots were used for different physicochemical assays as described earlier [21]. The freshly harvested whole sprouts containing cotyledons, hypocotyl, and roots were kept at −70 °C for 24 h before freeze-drying. The freeze-dried sprout samples were ground into powder using a commercial grinder (HIL-G-501, Hanil Co., Seoul, Korea) and filtered using a 100-mesh sieve.

### 4.4. Measurement of Moisture and Vitamin C Contents

The moisture content of soybean sprout was estimated following the method described earlier [17,21]. Five grams of freshly harvested sprouts were oven-dried (60 °C) to constant weight, and the moisture content was determined using the following equation:

Moisture content (%) = [(Wb − Wa)/Wb] × 100,(1)

where Wb and Wa are the weights of sprouts before and after oven-drying.

The vitamin C analysis of soybean sprouts was carried out following the method of AOAC [56]. Five grams of sample powder was mixed in 7.5 mL 3% metaphosphoric acid solution and homogenized (AM-8, Nihonseike Kaisha, Tokyo, Japan). The mixture was filtered and made the final volume up to 12.5 mL. Six milliliters of the reaction mixture was titrated with 0.025% of 2,6-dichloroindophenol. In this reaction, vitamin C content in the mixture was oxidized, and the indophenol dye was reduced to a colorless compound.

### 4.5. Color Measurement

The color values of samples were determined following the Hunter’s color measurement system as described earlier [57]. The ‘L’ (lightness), ‘a’ (redness), and ‘b’ (yellowness) values were measured using a Chroma Meter (CR-300, Minolta Corp., Tokyo, Japan). A calibration plate (Minolta Corp.; YCIE = 94.5, XCIE = 0.3160, YCIE = 0.330) and a standard plate (Hunter Associates Laboratory Inc., Reston, VA, USA; ‘L’ = 97.51, ‘a’ = −0.18, ‘b’ = 1.67) were considered for standardizing the instrument with D65 illuminant.

### 4.6. Determination of Free Amino Acid

The free amino acid profile was determined following the procedure described by Je et al. [58]. Sprout powder (1.5 g) was homogenized (12,000 rpm, 2 min) with 10 mL of ice-cold 6% (*v*/*v*) perchloric acid in an ice bath using an ACE homogenizer (Nissei AM-7, Nihonseikei Kaisha Ltd., Tokyo, Japan) and then incubated for 30 min in ice before centrifugation (5000 rpm, 15 min). The supernatant was filtered through a filter paper (Whatman No. 41). The filtrate pH (7.0) was adjusted using KOH solution (33%, *w*/*v*), and centrifuged (5000 rpm, 10 min) to remove the precipitate of potassium perchlorate. The pH of the supernatant was adjusted to 2.2 with a 10 M HCl, and the final volume was brought to 50 mL with distilled water. A sample aliquot (2 mL) was mixed with 1 mL of lithium citrate buffer (pH 2.2). Then, free amino acids were analyzed using an automatic amino acid analyzer (Biochrom-20, Pharmacia Biotech Co., Uppsala, Sweden).

### 4.7. Determination of Mineral Content

The mineral content was determined using an inductively coupled plasma atomic emission spectrometer (ICP AES: Varian Vista, Victoria, Australia) following the procedure of Skujins [59]. Sprout powder (0.5 g) was digested in a mixture of 65% HNO_3_ (15.0 mL) and 35% H_2_O_2_ (2 mL). An equal volume of distilled water was added to dilute the mixture. The mineral content of the samples was estimated after calibrating the ICP AES with known standards.

### 4.8. Determination of Isoflavone Content

The amounts of isoflavones in the samples were determined using a High-Performance Liquid Chromatography (HPLC) system (Prostar 230, Varian Co., California, USA) by following the method described by Jiao et al. [60]. The HPLC system consisted of a Nova-Pak C18 reversed-phased column and Adsorbosphere C18 direct-connect guard column (Waters Co., Milford, MA, USA) and UV detector (Varian Co., California, USA). Two hundred micrograms of sample powder were extracted with 6 mL methanol (80%) using an ultrasonic-assisted method at 40 °C for 30 min. The mixture was centrifuged (3000 rpm, 10 min), and the supernatant was filtered through a membrane filter (0.45 µm, Millipore, Bedford, MA, USA). The filtrate was used in HPLC for isoflavone analysis. The HPLC system was set as follows: flow rate 1 mL/min; the mobile phase: solvent A—aqueous acetic acid (0.1%), and solvent B—acetic acid in acetonitrile (0.1%). The HPLC was run at a gradient of 13–35% for 52 min, and the oven temperature was set to 35 °C. The injection volume was 20 µL. The eluted isoflavones were detected at 260 nm. Each peak was identified by the retention time and the characteristic UV spectrum in comparison with the corresponding standards.

### 4.9. Extraction of Sample

One gram of sample powder was extracted with 10 mL of absolute methanol using a shaking incubator (250 rpm, 25 °C) for 2 h. The mixture was centrifuged (3000 rpm, 15 min) and the supernatant was filtered through a syringe filter (0.2 µm). The filtrate extract was used for antioxidant assays.

### 4.10. Determination of DPPH Radical Scavenging Activity

The DPPH free radical scavenging potential of the samples was determined as described in earlier reports [61,62]. Equal volumes of sample extract (100 μL) and freshly prepared 0.05% (*w*/*v*) methanolic solution of DPPH (100 μL) were put into a 96-well microplate and allowed to react at room temperature for 30 min under dark condition. After 30 min, the absorbance values of the reaction mixtures were measured at 517 nm using a spectrophotometer (Multiskan GO, Thermo Fisher Scientific Oy, Vantaa, Finland). The free radical scavenging potential was calculated as follows.

Scavenging potential (%) = [1 − (S − So)/(C − Co)] × 100,(2)

where S, So, C, and Co are the absorbance values of the sample and DPPH, sample and methanol, methanol and DPPH, and methanol, respectively.

### 4.11. Determination of Superoxide Dismutase (SOD)-Like Activity

The SOD-like activities of the sample extracts were estimated following the methods described by Debnath et al. [63] and Adhikari et al. [64]. Reaction mixtures of different samples were prepared by mixing 1300 μL Tris-HCl buffer (50 mM Tris, 10 mM EDTA, pH 8.5), 100 μL 7.2 mM pyrogallol, and 100 μL sample extracts and then incubated at room temperature for 10 min under dark condition. After the incubation, the reaction was terminated by adding 50 μL 1 N HCl into the mixture. The amounts of pyrogallol oxidized during the reaction were determined by measuring the absorbance values of the mixtures at 420 nm using a microplate spectrophotometer (Multiskan GO, Thermo Fisher Scientific). The SOD-like activity was determined using the following equation.

SOD-like activity (%) = [1 − (S − So)/S] ×100,(3)

where S and So are the absorbance of solution without sample and absorbance of solution with sample, respectively.

### 4.12. Determination of Total Polyphenol

The total polyphenol content (TPC) of samples was measured according to the Folin–Ciocalteau method [65] as described by Dhungana et al. [66]. The sample extracts (50 μL) and 2% (*w*/*v*) aqueous Na_2_CO_3_ (1000 μL) were mixed in microtubes and kept at room temperature for 3 min. Then, 1 N Folin–Ciocalteau reagent (50 μL) was put into the mixture and incubated at room temperature for 30 min under dark condition. The absorbance value of reaction mixtures was read at 750 nm using a microplate spectrophotometer (Multiskan GO; Thermo Fisher Scientific). A calibration curve was plotted using gallic acid (GA). The TPC content in the samples was estimated as GA equivalent (GAE).

### 4.13. Flavonoid Content Analysis

The flavonoid content was determined following the methods described earlier [66,67]. The sample extracts (100 μL), absolute methanol (500 μL), 10% AlCl_3_ (50 μL), 1 M HCl (50 μL), and distilled water (300 μL) were mixed in microtubes and incubated as in the TPC determination method. After 30 min of incubation, the absorbance values of the reaction mixtures were measured at 510 nm using a microplate spectrophotometer (Multiskan GO; Thermo Fischer Scientific). The calibration curve for flavonoid determination was created using quercetin as a standard. The flavonoid content was expressed as a quercetin equivalent (QE).

### 4.14. Statistical Analysis

Statistical analyses were carried out by analysis of variance using SAS 9.4 (SAS Institute, Cary, NC, USA). The significant differences between samples were determined at 5% probability using Tukey’s test. The average values of three replicates are reported unless otherwise mentioned.

## 5. Conclusions

The effects of Pu-erh tea extracts on soybean sprouts were investigated considering the yield and nutritional values of the sprouts. The yield, antioxidant potentials, and contents of vitamin C, total free amino acid, total mineral, isoflavone, total polyphenol, and flavonoid of the soybean sprouts treated with the tea extracts were higher than those of the control. Overall, the soaking of soybean seeds in 2% (*w*/*v)* Pu-erh tea extract could be an easy and efficient measure to enhance the yield and nutritional values of soybean sprouts.

## Figures and Tables

**Table 1:** Effect of Pu-erh tea extracts on yield, moisture, and vitamin C content of soybean sprouts grown for 6 days.

Sample ^1^	Total Weight (g)	Moisture (%)	Vitamin C (mg/100 g Fresh Weight)
Control	6843 ± 38c ^2^ (100.0%)	87.11 ± 1.02a	16.00 ± 0.11c
PE-1	7492 ± 32b (109.5%)	87.12 ± 0.08a	16.90 ± 0.21b
PE-2	7998 ± 50a (116.8%)	87.08 ± 1.12a	18.07 ± 0.32a
PE-3	8005 ± 45a (116.9%)	87.00 ± 0.80a	18.09 ± 0.20a

^1^ Samples are defined in the Materials and Methods section.^2^ Values are expressed as mean ± standard deviation of three replicates. Values followed by different letters (a, b and c) in the same column indicate significant difference (*p* < 0.05, ANOVA, Tukey test).

**Table 2:** Hunter’s color values of soybean sprouts grown after seed-soaking in different concentrations of Pu-erh tea extracts.

Sample ^1^	Color Value ^2^		
	L	a	b
Control	71.90 ± 0.06c ^3^	−0.81 ± 0.07a	17.73 ± 1.12c
PE-1	75.39 ± 0.85a	−1.41 ± 0.09c	22.32 ± 0.50a
PE-2	75.73 ± 0.58a	−1.12 ± 0.06b	22.36 ± 0.22a
PE-3	73.38 ± 0.41b	−1.17 ± 0.14b	21.02 ± 0.26b

^1^ Samples are defined in the Materials and Methods section. ^2^ L, lightness (100, white; 0, black); a, redness (−, green; +, red); b, yellowness (−, blue; +, yellow). ^3^ Values are expressed as mean ± standard deviation of three replicates. Values followed by different letters (a, b, and c) in the same column are significantly different (*p* < 0.05, ANOVA, Tukey test).

**Table 3:** Free amino acid composition (mg/g of dry weight) of soybean sprouts grown after seed-soaking in different concentrations of Pu-erh tea extracts. PE-1, PE-2, and PE-3: 1%, 2%, and 3% (*w*/*v*) Pu-erh tea extract contractions, respectively.

Amino Acid	Sample ^1^			
	Control	PE-1	PE-2	PE-3
Essential Amino Acid				
l-Threonine	1.36 ± 0.02d ^2^	1.65 ± 0.01a	1.50 ± 0.01c	1.61 ± 0.01b
l-Valine	2.26 ± 0.02d	2.86 ± 0.03a	2.49 ± 0.02c	2.79 ± 0.03b
l-Methionine	0.33 ± 0.01b	0.36 ± 0.01a	0.36 ± 0.01a	0.35 ± 0.01b
l-Isoleucine	1.33 ± 0.01c	1.61 ± 0.04a	1.50 ± 0.02b	1.57 ± 0.02a
l-Leucine	0.91 ± 0.02c	1.03 ± 0.01a	0.99 ± 0.01b	1.01 ± 0.01ab
l-Phenylalanine	1.79 ± 0.03b	1.96 ± 0.02a	1.82 ± 0.01b	1.95 ± 0.01a
l-Lysine	2.64 ± 0.03c	2.89 ± 0.02a	2.77 ± 0.01b	2.74 ± 0.02b
l-Histidine	2.71 ± 0.02d	3.24 ± 0.06b	3.14 ± 0.03c	3.31 ± 0.02a
Subtotal	13.33	15.60	14.57	15.33
Non-Essential Amino Acid				
l-Asparitic acid	0.88 ± 0.02c	0.97 ± 0.03b	1.03 ± 0.01a	1.05 ± 0.02a
L-Serine	2.96 ± 0.03c	3.41 ± 0.02a	3.15 ± 0.01b	3.39 ± 0.03a
l-Glutamic acid	5.29 ± 0.07d	5.81 ± 0.05b	5.57 ± 0.06c	6.06 ± 0.02a
Glycine	0.30 ± 0.01b	0.36 ± 0.02a	0.30 ± 0.01b	0.32 ± 0.03ab
l-Alanine	1.79 ± 0.01d	2.16 ± 0.02b	2.09 ± 0.02c	2.20 ± 0.01a
l-Tyrosine	0.28 ± 0.02b	0.31 ± 0.02b	0.38 ± 0.01a	0.30 ± 0.03b
l-Arginine	15.61 ± 0.16b	14.78 ± 0.12d	17.06 ± 0.07a	15.27 ± 0.05c
Proline	1.05 ± 0.01b	1.12 ± 0.02a	0.97 ± 0.01c	1.06 ± 0.01b
Subtotal	28.16	28.92	30.55	29.65
Essential to Non-Essential Ratio	0.47	0.54	0.48	0.52
Other Amino Acid				
*O*-Phospho-l-serine	0.16 ± 0.02a	0.18 ± 0.01a	0.17 ± 0.01a	0.17 ± 0.02a
Taurine	ND ^3^	ND	ND	ND
*O*-Phospho ethanol amine	ND	ND	ND	ND
Urea	2.37 ± 0.03b	2.69 ± 0.04a	2.18 ± 0.02c	2.33 ± 0.01b
l-Sarcosine	ND	ND	ND	ND
l-α-Amino asipic acid	0.36 ± 0.02b	0.39 ± 0.01b	0.44 ± 0.02a	0.44 ± 0.01a
L-Citrulline	0.18 ± 0.01a	0.19 ± 0.01a	0.21 ± 0.03a	0.20 ± 0.02a
l-α-Amino-n-butyric acid	0.08 ± 0.02a	0.09 ± 0.02a	0.10 ± 0.01a	0.09 ± 0.01a
l-Cystine	ND	ND	ND	ND
Cystathionine	ND	ND	ND	ND
β-Alanine	0.36 ± 0.01b	0.48 ± 0.03a	0.37 ± 0.02b	0.43 ± 0.03a
d, l-β-Amino isobutyric acid	0.16 ± 0.02a	0.18 ± 0.02a	0.18 ± 0.02a	0.19 ± 0.01a
γ-Amino-n-butyric acid	0.53 ± 0.01b	0.67 ± 0.03a	0.70 ± 0.03a	0.66 ± 0.02a
Ethanolamin	0.21 ± 0.02a	0.21 ± 0.01a	0.20 ± 0.02a	0.21 ± 0.01a
Hydroxylysine	ND	ND	ND	ND
l-Ornithine	0.04 ± 0.01c	0.12 ± 0.02b	0.21 ± 0.01a	0.19 ± 0.01a
1-Methyl-l-histidine	ND	ND	ND	ND
3-Methyl-l-histidine	ND	ND	ND	ND
l-Anserine	ND	ND	ND	ND
l-Carnosine	ND	ND	ND	ND
Hydroxy proline	0.19 ± 0.04a	0.21 ± 0.03a	0.17 ± 0.02a	0.21 ± 0.03a
Subtotal	4.64	5.41	4.93	5.12
**Total Free Amino Acid**	**46.13**	**49.93**	**50.05**	**50.10**

^1^ Samples are defined in the Materials and Methods section. ^2^ Values are expressed as mean ± standard deviation of two replicates. Values followed by different letters (a, b, c, and d) in the same row are significantly different (*p* < 0.05, ANOVA, Tukey test). ^3^ Non-detected.

**Table 4:** Mineral contents (mg/kg of dry weight) of soybean sprouts grown after seed-soaking in different concentrations of Pu-erh tea extracts.

Element	Sample ^1^			
	Control	PE-1	PE-2	PE-3
Ca	1624.86 ± 28.28c ^2^	1636.74 ± 27.94c	1942.67 ± 26.53a	1864.39 ± 33.02b
Cu	39.44 ± 0.15b	41.66 ± 0.90a	34.19 ± 0.22c	29.70 ± 0.56d
Fe	84.08 ± 1.52c	92.96 ± 0.67b	99.55 ± 1.13a	100.69 ± 1.58a
K	16845.74 ± 10.92d	17557.39 ± 11.62b	17043.15 ± 11.03c	18180.18 ± 21.13a
Mg	1663.33 ± 14.04b	1683.26 ± 13.10b	1777.44 ± 14.40a	1778.53 ± 16.48a
Mn	22.42 ± 0.31a	20.66 ± 0.30c	21.51 ± 0.26b	22.24 ± 0.33a
Na	284.57 ± 0.33d	330.58 ± 2.45c	376.18 ± 0.59a	369.00 ± 3.87b
Zn	55.67 ± 0.08a	51.79 ± 0.07c	55.91 ± 0.22a	53.85 ± 0.30b
**Total**	**20,620.11**	**21,415.04**	**21,350.6**	**22,398.58**

^1^ Samples are defined in the Materials and Methods section. ^2^ Values are expressed as mean ± standard deviation of two replicates. Values followed by different letters (a, b, c, and d) in the same row are significantly different (*p* < 0.05, ANOVA, Tukey test).

**Table 5:** Isoflavone content of soybean sprouts grown after seed-soaking in different concentrations of Pu-erh tea extracts.

Sample ^1^	Isoflavone Content (mg/kg dry weight)						
	Daidzin	Daidzein	Genistin	Glycitin	Glycitein	Genistein	Total
Control	321.41 ± 3.21c ^2^	19.31 ± 3.71b	281.11 ± 3.52c	88.91 ± 5.53c	10.11 ± 1.72d	36.11 ± 2.21a	756.95
PE-1	351.23 ± 5.21b	25.20 ± 1.21a	299.81 ± 5.71b	95.21 ± 3.62b	12.91 ± 3.01c	35.20 ± 3.00a	819.57
PE-2	399.91 ± 6.91a	25.32 ± 2.71a	350.21 ± 8.12a	99.21 ± 2.72a	14.91 ± 1.12b	36.51 ± 2.41a	926.08
PE-3	401.21 ± 2.10a	24.11 ± 1.62a	359.44 ± 6.94a	102.02 ± 5.43a	15.71 ± 1.62a	37.12 ± 2.72a	939.59

^1^ Samples are defined in the Materials and Methods section. ^2^ Values are expressed as the mean ± standard deviation of three replicates. Values followed by different letters (a, b, c, and d) in the same column are significantly different (*p* < 0.05, ANOVA, Tukey test).

**Table 6:** 1,1-Diphenly-2-picrylhydrazyl (DPPH) and superoxide dismutase (SOD)-like activities and total polyphenol and flavonoid contents of soybean sprouts grown after seed-soaking in different concentrations of Pu-erh tea extracts.

Sample ^1^	% Inhibition		Total Polyphenol (μg GAE ^2^/g dry weight)	Flavonoid (μg QE ^3^/g dry weight)
	DPPH	SOD-Like Activity		
Control	80.44 ± 2.91c ^4^	79.73 ± 1.51c	348.20 ± 0.36c ^4^	90.52 ± 2.37b
PE-1	84.16 ± 2.72b	85.37 ± 0.70a	423.23 ± 3.55a	107.50 ± 3.41a
PE-2	87.34 ± 0.84a	82.08 ± 0.29b	406.83 ± 1.85b	109.40 ± 5.32a
PE-3	87.24 ± 1.10a	83.76 ± 0.96b	404.35 ± 2.06b	105.86 ± 4.66a

^1^ Samples are defined in the Materials and Methods section. ^2^ GAE: gallic acid equivalents. ^3^ QE: quercetin equivalents. ^4^ Values are expressed as mean ± standard deviation of three replicates. Values followed by different letters (a, b, and c) in the same column are significantly different (*p* < 0.05, ANOVA, Tukey test).

## Supplementary Materials

The following are available online, Table S1: Analysis of variance for yield, moisture content, vitamin C content, color value, total free amino acid content, total mineral, total isoflavone, DPPH, SOD-like activity, total polyphenol content, and flavonoid content of Pu-erh tea extract-treated soybean sprouts grown for 6 days.

## References

[1] Kumari S., Krishnan V., Sachdev A. (2015). Impact of soaking and germination durations on antioxidants and anti-nutrients of black and yellow soybean (*Glycine max*. L) varieties. J. Plant Biochem. Biotechnol..

[2] Fernandez-Orozco R., Frias J., Zielinski H., Piskula M.K., Kozlowska H., Vidal-Valverde C. (2008). Kinetic study of the antioxidant compounds and antioxidant capacity during germination of *Vigna radiata* cv. Emmerald, *Glycine max* cv. Jutro and *Glycine max* cv. Merit. Food Chem..

[3] Cevallos-Casals B.A., Cisneros-Zevallos L. (2010). Impact of germination on phenolic content and antioxidant activity of 13 edible seed species. Food Chem..

[4] Paucar-Menacho L.M., Berhow M.A., Mandarino J.M.G., Chang Y.K., Mejia E.G. (2010). de Effect of time and temperature on bioactive compounds in germinated Brazilian soybean cultivar BRS 258. Food Res. Int..

[5] Silva L.R., Pereira M.J., Azevedo J., Gonçalves R.F., Valentão P., de Pinho P.G., Andrade P.B. (2013). *Glycine max* (L.) Merr., *Vigna radiata* L. and *Medicago sativa* L. sprouts: A natural source of bioactive compounds. Food Res. Int..

[6] Spanier A.M., Shahidi F., Parliment T.H., Mussinan C., Ho C.-T., Tratras Contis E., Kayahara H., Tsukahara K., Tatai T., Spanier A.M., Shahidi F., Parliment T.H., Mussinan C., Ho C.-H., Contis C.T. (2001). Flavor, health and nutritional quality of pre-germinated brown rice. Food Flavors and Chemistry: Advances of the New Millennium.

[7] Wang X., Yang R., Jin X., Shen C., Zhou Y., Chen Z., Gu Z. (2016). Effect of supplemental Ca^2+^ on yield and quality characteristics of soybean sprouts. Sci. Hortic..

[8] Yang H., Gao J., Yang A., Chen H. (2015). The ultrasound-treated soybean seeds improve edibility and nutritional quality of soybean sprouts. Food Res. Int..

[9] Mody I., De Koninck Y., Otis T.S., Soltesz I. (1994). Bridging the cleft at GABA synapses in the brain. Trends Neurosci..

[10] Oh C.-H., Oh S.-H. (2004). Effects of ferminated brown rice extracts with enhanced levels of GABA on cancer cell proliferation and apoptosis. J. Med. Food.

[11] Dhakal R., Bajpai V.K., Baek K.-H. (2012). Production of GABA (γ-aminobutyric acid) by microorganisms: A review. Braz. J. Microbiol..

[12] Okada T., Sugishita T., Murakami T., Murai H., Saikusa T., Horino T., Onoda A., Kajimoto O., Takahashi R., Takahashi T. (2000). Effect of the defatted rice germ enriched with GABA for sleeplessness, depression, autonomic disorder by oral administration. Nippon. Shokuhin. Kagaku. Kogaku. Kaishi..

[13] Reeds P.J. (2000). Dispensable and indispensable amino acids for humans. J. Nutr..

[14] Chen Y., Chang S.K.C. (2015). Macronutrients, phytochemicals, and antioxidant activity of soybean sprout germinated with or without light exposure. J. Food Sci..

[15] Yun J., Li X., Fan X., Li W., Jiang Y. (2013). Growth and quality of soybean sprouts (*Glycine max* L. Merrill) as affected by gamma irradiation. Radiat. Phys. Chem..

[16] Zou T., Xu N., Hu G., Pang J., Xu H. (2014). Biofortification of soybean sprouts with zinc and bioaccessibility of zinc in the sprouts. J. Sci. Food Agric..

[17] Lee S.-J., Ahn J.-K., Khanh T.-D., Chun S.-C., Kim S.-L., Ro H.-M., Song H.-K., Chung I.-M. (2007). Comparison of isoflavone concentrations in soybean (*Glycine max* (L.) Merrill) sprouts grown under two different light conditions. J. Agric. Food Chem..

[18] Algar E., Ramos-Solano B., García-Villaraco A., Saco Sierra M.D., Martín Gómez M.S., Gutiérrez-Mañero F.J. (2013). Bacterial bioeffectors modify bioactive profile and increase isoflavone content in soybean sprouts (*Glycine max* var Osumi). Plant. Foods Hum. Nutr..

[19] Choi H.D., Kim S.S., Kim S.R., Lee B.Y. (2000). Effect of irrigating solutions on growth and rot of soybean sprouts. Korean J. Food Sci. Technol..

[20] Choi S.D., Kim Y.H., Nam S.H., Shon M.Y., Choi J. (2003). Changes in major taste components of soybean sprouts germinated with extract of Korean *Panax ginseng*. Korean J. Life Sci..

[21] Kim I.-D., Dhungana S.K., Park Y.-S., Kim D., Shin D.-H. (2017). Persimmon fruit powder may substitute Indolbi, a synthetic growth regulator, in soybean sprout cultivation. Molecules.

[22] Nakamura S., Kaneko K., Mitsui T., Ohtsubo K. (2020). Evaluation of the palatability and biofunctionality of brown rice germinated in red onion solution. Cereal Chem..

[23] Huang S., Yang J., Sun Y., Tang Y., Yang H., Liu C. (2015). Effects of 6-benzylaminopurine on soybean sprouts growth and determination of its residue. J. South. Agric..

[24] Chaikina E.L., Gerasimenko N.I., Dega L.A., Vashchenko A.P., Anisimov M.M. (2009). Effect of ethanol metabolites of seersucker kelp *Costaria costata* on growth of soybean sprouts. Russ. Agric. Sci..

[25] Kwak H.-S., Dhungana S.K., Kim I.-D., Shin D.-H. (2017). Effect of lacquer (*Toxicodendron vernicifluum*) extract on yield and nutritional value of soybean sprouts. Korean J. Food Sci. Technol..

[26] Lee L.K., Foo K.Y. (2013). Recent advances on the beneficial use and health implications of Pu-Erh tea. Food Res. Int..

[27] Choi S.-H., Kim I.-D., Dhungana S.K., Kim D.-G. (2018). Comparison of quality characteristic and antioxidant potential of cultivated Pu-erh and Gushu Pu-erh tea extracts at two temperatures. J. Pure Appl. Microbiol..

[28] Choi S.-H., Kim I.-D., Dhungana S.K., Park K. (2019). Effect of extraction temperature on physicochemical constituents and antioxidant potentials of Pu-erh tea. Korean J. Food Sci. Technol..

[29] Lintschinger J., Fuchs N., Moser J., Kuehnelt D., Goessler W. (2000). Selenium-enriched sprouts. A raw material for fortified cereal-based diets. J. Agric. Food Chem..

[30] McKenzie J.S., Jurado J.M., de Pablos F. (2010). Characterisation of tea leaves according to their total mineral content by means of probabilistic neural networks. Food Chem..

[31] Udomkun P., Ilukor J., Mockshell J., Mujawamariya G., Okafor C., Bullock R., Nabahungu N.L., Vanlauwe B. (2018). What are the key factors influencing consumers’ preference and willingness to pay for meat products in Eastern DRC?. Food Sci. Nutr..

[32] Park M., Kim D., Kim B., Nahmgoong B. (1995). Studies on pollution-free soybean sprout production and circulation market improvement. Korea Soybean Dig..

[33] Nikmaram N., Dar B., Roohinejad S., Koubaa M., Barba F.J., Greiner R., Johnson S.K. (2017). Recent advances in γ -aminobutyric acid (GABA) properties in pulses: An overview. J. Sci. Food Agric..

[34] Xu N., Zou T., Pang J.W., Hu G.L. (2012). Effects of exogenous zinc soaking on the seed germination, sprout growth of soybean and zinc accumulation in the sprouts. Soybean Sci..

[35] White P.J., Broadley M.R. (2009). Biofortification of crops with seven mineral elements often lacking in human diets - iron, zinc, copper, calcium, magnesium, selenium and iodine. New Phytol..

[36] Houston M.C., Harper K.J. (2008). Potassium, magnesium, and calcium: Their role in both the cause and treatment of hypertension. J. Clin. Hypertens..

[37] Ganz T. (2013). Systemic iron homeostasis. Physiol. Rev..

[38] MacDiarmid C.W. (2000). Zinc transporters that regulate vacuolar zinc storage in *Saccharomyces cerevisiae*. EMBO J..

[39] Jung W., Yu O., Lau S.-M.C., O’Keefe D.P., Odell J., Fader G., McGonigle B. (2000). Identification and expression of isoflavone synthase, the key enzyme for biosynthesis of isoflavones in legumes. Nat. Biotechnol..

[40] Yoon G.-A., Park S. (2014). Antioxidant action of soy isoflavones on oxidative stress and antioxidant enzyme activities in exercised rats. Nutr. Res. Pract..

[41] Setchell K.D.R., Cassidy A. (1999). Dietary isoflavones: Biological effects and relevance to human health. J. Nutr..

[42] Messina M., Gugger E.T., Alekel D.L., Wildman R.E. (2001). Soy protein, soybean isoflavones and bone health: A review of the animal and human data. Handbook of Nutraceuticals and Functional Food.

[43] Demonty I., Lamarche B., Jones P.J.H. (2003). Role of isoflavones in the hypocholesterolemic effect of soy. Nutr. Rev..

[44] Prakash D., Upadhyay G., Singh B.N., Singh H.B. (2007). Antioxidant and free radical-scavenging activities of seeds and agri-wastes of some varieties of soybean (*Glycine max*). Food Chem..

[45] Santos J.H., Hunakova L., Chen Y., Bortner C., Van Houten B. (2003). Cell sorting experiments link persistent mitochondrial DNA damage with loss of mitochondrial membrane potential and apoptotic cell death. J. Biol. Chem..

[46] Mishra S., Jha A.B., Dubey R.S. (2011). Arsenite treatment induces oxidative stress, upregulates antioxidant system, and causes phytochelatin synthesis in rice seedlings. Protoplasma..

[47] Srivastava S., Dubey R.S. (2011). Manganese-excess induces oxidative stress, lowers the pool of antioxidants and elevates activities of key antioxidative enzymes in rice seedlings. Plant. Growth Regul..

[48] Zhang H.-M., Wang C.-F., Shen S.-M., Wang G.-L., Liu P., Liu Z.-M., Wang Y.-Y., Du S.-S., Liu Z.-L., Deng Z.-W. (2012). Antioxidant phenolic compounds from Pu-erh tea. Molecules.

[49] Chen M., Zhu Y., Zhang H., Wang J., Liu X., Chen Z., Zheng M., Liu B. (2017). Phenolic compounds and the biological effects of Pu-erh teas with long-term storage. Int. J. Food Prop..

[50] Griffiths G., Trueman L., Crowther T., Thomas B., Smith B. (2002). Onions—A global benefit to health. Phytother. Res..

[51] Gennaro L., Leonardi C., Esposito F., Salucci M., Maiani G., Quaglia G., Fogliano V. (2002). Flavonoid and carbohydrate contents in tropea red onions: Effects of homelike peeling and storage. J. Agric. Food Chem..

[52] Rice-evans C.A., Miller N.J., Bolwell P.G., Bramley P.M., Pridham J.B. (1995). The relative antioxidant activities of plant-derived polyphenolic flavonoids. Free Radic. Res..

[53] Yang R., Guo L., Zhou Y., Shen C., Gu Z. (2015). Calcium mitigates the stress caused by ZnSO_4_ as a sulphur fertilizer and enhances the sulforaphane formation of broccoli sprouts. RSC Adv..

[54] Park K.Y., Yun H.T., Moon J.K., Ku J.H., Hwang J.J., Lee S.H., Seung Y.K., Ryu Y.H., Chung W.K., Lee Y.H. (2000). A new soybean cultivar for sprout with good storability and disease resistance, ‘Sowonkong’. Korean J. Breed..

[55] Tianlu M., Wenju Z. (1996). The evolution and distribution of genus Camellia. Acta Bot. Yunnanica..

[56] AOAC Association of Official Analytical Chemists (1990). Official Methods of Analysis of the Association of Official Analytical Chemists.

[57] Kim I.-D., Lee J.-W., Kim S.-J., Cho J.-W., Dhungana S.K., Lim Y.-S., Shin D.-H. (2014). Exogenous application of natural extracts of persimmon (*Diospyros kaki* Thunb.) can help in maintaining nutritional and mineral composition of dried persimmon. Afr. J. Biotechnol..

[58] Je J.-Y., Park P.-J., Jung W.-K., Kim S.-K. (2005). Amino acid changes in fermented oyster (*Crassostrea gigas*) sauce with different fermentation periods. Food Chem..

[59] Skujins S. (1998). Handbook for ICP-AES (Varian-Vista). A Short Guide to Vista Series ICP-AES Operation.

[60] Jiao C., Yang R., Zhou Y., Gu Z. (2016). Nitric oxide mediates isoflavone accumulation and the antioxidant system enhancement in soybean sprouts. Food Chem..

[61] Dhungana S.K., Kim B.-R., Son J.-H., Kim H.-R., Shin D.-H. (2015). Comparative study of *CaMsrB2* gene containing drought-tolerant transgenic rice (*Oryza sativa* L.) and non-transgenic counterpart. J. Agron. Crop. Sci..

[62] Blois M.S. (1958). Antioxidant determinations by the use of a stable free radical. Nature.

[63] Debnath T., Park P.-J., Deb Nath N.C., Samad N.B., Park H.W., Lim B.O. (2011). Antioxidant activity of *Gardenia jasminoides* Ellis fruit extracts. Food Chem..

[64] Adhikari B., Dhungana S.K., Kim I.-D., Shin D.-H. (2020). Effect of foliar application of potassium fertilizers on soybean plants under salinity stress. J. Saudi Soc. Agric. Sci..

[65] Singleton V.L., Orthofer R., Lamuela-Raventós R.M., Wilchek M., Bayer E.A. (1999). [14] Analysis of total phenols and other oxidation substrates and antioxidants by means of folin-ciocalteu reagent. Methods in enzymology.

[66] Dhungana S.K., Kim I.-D., Kwak H.-S., Shin D.-H. (2016). Unraveling the effect of structurally different classes of insecticide on germination and early plant growth of soybean [*Glycine max* (L.) Merr.]. Pestic. Biochem. Physiol..

[67] Zhishen J., Mengcheng T., Jianming W. (1999). The determination of flavonoid contents in mulberry and their scavenging effects on superoxide radicals. Food Chem..

